# Birth outcomes in single motherhood ‘by choice’: a register study of children conceived through medically assisted reproduction

**DOI:** 10.1093/ije/dyag172

**Published:** 2026-09-23

**Authors:** Alina Pelikh, Hanna Remes, Mine Kühn, Pekka Martikainen, Alice Goisis

**Affiliations:** Centre for Longitudinal Studies, University College London, London, United Kingdom; Helsinki Institute for Demography and Population Health, University of Helsinki, Helsinki, Finland; Max Planck – University of Helsinki Center for Social Inequalities in Population Health, Helsinki, Finland; School of Social and Behavioral Sciences, Tilburg University, Tilburg, The Netherlands; Max Planck Institute for Demographic Research, Rostock, Germany; Einstein Center Population Diversity, Berlin, Germany; Helsinki Institute for Demography and Population Health, University of Helsinki, Helsinki, Finland; Max Planck – University of Helsinki Center for Social Inequalities in Population Health, Helsinki, Finland; Max Planck Institute for Demographic Research, Rostock, Germany; Centre for Longitudinal Studies, University College London, London, United Kingdom

**Keywords:** birth outcomes, low birthweight, preterm birth, medically assisted reproduction, single motherhood, single mothers ‘by choice’

## Abstract

**Background:**

Single mothers ‘by choice’ who conceive through medically assisted reproduction (MAR) are a growing and understudied group. We examined how their infants’ birth outcomes compare with other family types.

**Methods:**

Using Finnish population register data (1996–2020), we examined five birth outcomes among first-born children: low birthweight (LBW), preterm birth, small for gestational age (SGA), gestational-age-adjusted birthweight, and gestational age. We compared children of (i) single MAR mothers (*n* = 2230) with those of (ii) single mothers who conceived spontaneously (SC; *n* = 36 352); (iii) partnered MAR mothers (*n* = 39 350); and (iv) partnered SC mothers (*n* = 451 295). Regression models adjusted for maternal age, education, income, smoking, mental health, and multiplicity.

**Results:**

In unadjusted analyses, children of single MAR mothers (reference) had higher risks of LBW and preterm and lower gestational age than those of partnered and single SC mothers [e.g. odds ratio (OR) for LBW = 0.51; 95% confidence interval (CI) 0.44–0.60 and 0.69; 95% CI 0.59–0.81, respectively]. After adjustments, differences relative to the partnered SC group largely attenuated (e.g. OR for LBW = 1.01; 95% CI 0.85–1.21), whereas outcomes were better than those of single SC and partnered MAR mothers (e.g. OR for LBW = 1.21; 95% CI 1.01–1.45 and 1.30; 95% CI 1.08–1.55, respectively). The relative advantage in SGA and gestational-age-adjusted birthweight also remained after adjustment.

**Conclusions:**

After accounting for sociodemographic characteristics and multiplicity, children of single mothers ‘by choice’ had birth outcomes comparable to those of partnered SC mothers and more favourable than those of single SC and partnered MAR mothers.

Key Messages

- This study provides novel population-level evidence on birth outcomes among children born to single mothers ‘by choice’ who conceive through medically assisted reproduction (MAR), a growing and understudied group, by comparing them with children born to single and partnered mothers who conceived spontaneously (SC) or through MAR.
- After adjustment for maternal characteristics and multiple births, birth outcomes were comparable to those of partnered SC mothers and more favourable than those of single SC and partnered MAR mothers.
- The findings suggest that the elevated risks observed before adjustment are largely attributable to higher rates of multiple births in the single MAR group and unlikely to be related to family structure or MAR treatments per se.

## Introduction

Since the birth of the first IVF baby in 1978, over 10 million children worldwide have been conceived through assisted reproductive techniques (ART) [1]. As the use of infertility treatments increases globally, understanding its impact on children’s health outcomes is crucial. Studies on birth outcomes among children conceived through medically assisted reproduction [MAR; including ovulation induction (OI), artificial insemination (AI), and ART] have found higher risks of adverse perinatal outcomes, such as low birthweight (LBW) and preterm, compared to spontaneously conceived (SC) children [2–5]. A growing subgroup within this population is children born to single mothers ‘by choice’ (women who conceive through MAR without a partner) [6–8]. Existing studies often only distinguish between married and unmarried women [4, 5], but fail to look specifically at children born to single mothers through MAR. This is a significant knowledge gap not only because of the rapid increase of this group [9–12] but also because there is evidence that children born to single mothers face increased risk of poorer birth outcomes [13, 14], which can have long-term implications for children’s health, social outcomes, and public health costs [15–17]. It is important to determine whether this is also true for single mothers through MAR.

Single MAR mothers share traits with both single SC and partnered MAR mothers but also possess characteristics distinguishing them from both groups which makes it difficult to predict a priori what their children’s birth outcomes might look like. They may experience higher stress levels during conception and pregnancy due to lack of practical and emotional support of a partner or societal stigma associated with single motherhood [18, 19] (similar to single SC mothers) and stress associated with undergoing infertility treatments [20–24] (similar to partnered MAR mothers), potentially negatively impacting birth outcomes. In addition, women who undergo MAR are on average older [2, 4, 5] and tend to report poorer mental health prior to birth [23], factors that may further contribute to adverse birth outcomes [4, 5]. However, they often have advantageous socioeconomic characteristics similar to partnered MAR mothers, such as higher education and income levels [25–29], as well as better health behaviours [4, 5] (e.g. lower rates of pregnancy smoking) which could have protective effects. Two key factors further characterize single MAR mothers. First, they have strong fertility intentions, and their pregnancies are intended, a factor strongly associated with better outcomes [30]. Second, unlike partnered MAR mothers, single MAR mothers do not necessarily suffer from subfertility, which is found to be associated with worse birth outcomes [2–5]. These unique characteristics of single MAR mothers represent an opportunity to learn about the impact of MAR and single motherhood on children’s birth outcomes.

This study utilizes Finnish population register data (1996–2020) to analyse birth outcomes of single MAR mothers. This data contains rich socioeconomic and health indicators, allowing adjustment for confounding in the associations with birth outcomes. To disentangle the potential effects of single parenthood and MAR treatments, we compare outcomes with those of single SC, partnered MAR, and partnered SC mothers. Although single women gained access to public infertility treatment in 2006, in practice most underwent MAR in private clinics until 2017 due to prioritisation of medically infertile patients.

## Materials and methods

### Study population

We analysed first-born children in Finland between 1996 and 2020 (*n* = 564 582), focusing on first births for comparability, as MAR children are predominantly first-borns [2, 4, 5], which is typically associated with worse birth outcomes than higher-order parities [31]. After exclusions, the final sample comprised 529 227 live births, with 41 580 (7.8%) conceived through MAR (Fig. 1).

**Figure 1:**
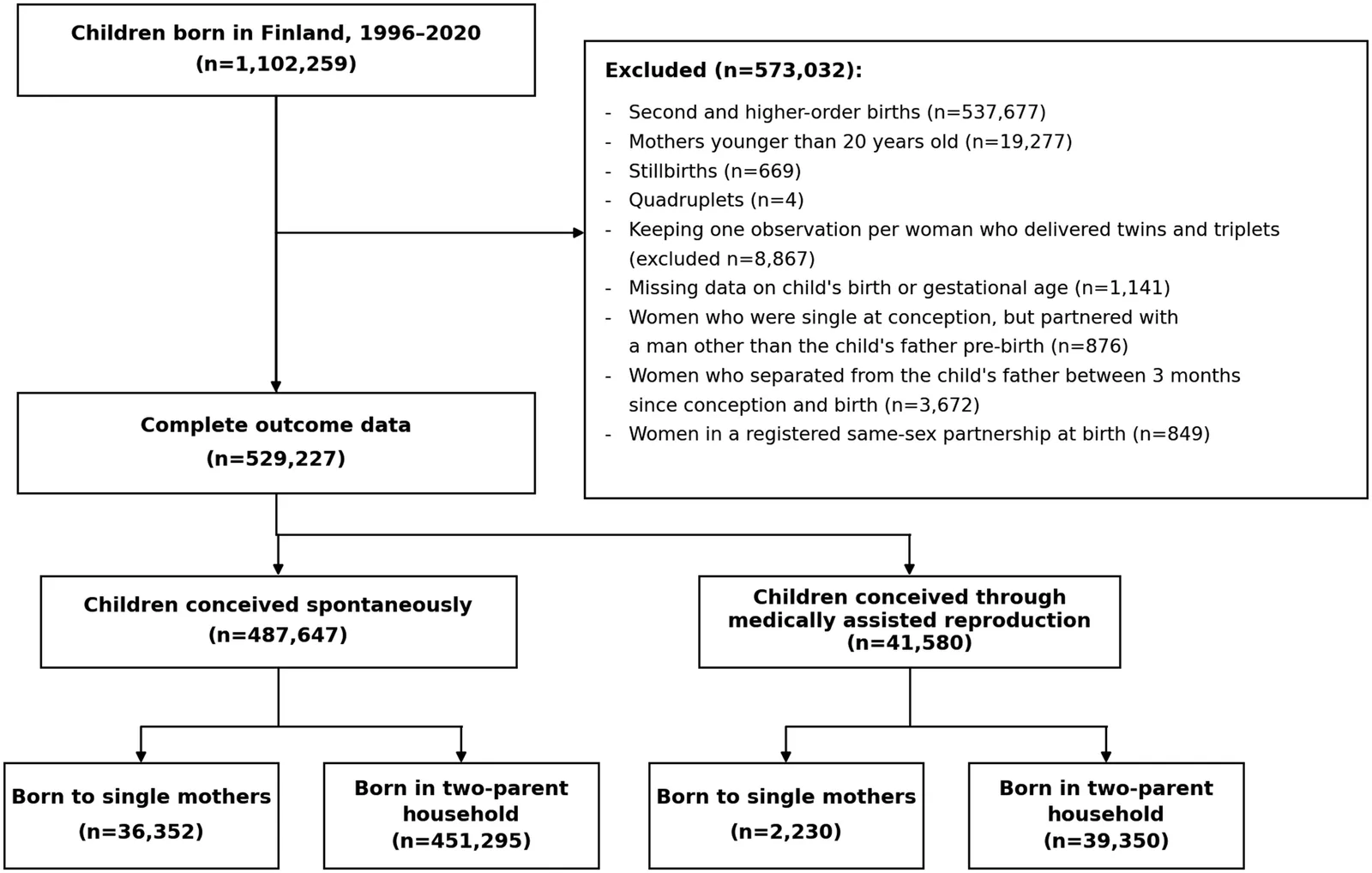
The figure shows the selection of the analytical sample from all births in Finland between 1996 and 2020. After applying exclusion criteria—including higher-order births, maternal age under 20, stillbirths, missing data, and partnership-related criteria—the final sample comprised 529 227 first births with complete data. These were classified by mode of conception (spontaneous conception or medically assisted reproduction) and maternal partnership status (single mother or two-parent household).

### Identifying single mothers ‘by choice’

We identified children born through MAR using birth register data, combined with records on infertility drug purchases (public and private sectors) and private clinic MAR treatments from the Social Insurance Institution and public MAR treatments from the national healthcare register (see Goisis [23], for further detail). MAR conception was defined by linking maternal treatment or drug purchase dates to birthdates.

We identified single mothers using Statistics Finland’s cohabitation data, which records the timing of moving in together, marriage, separation, divorce, and partner’s death. Women were classified as single mothers if they were not cohabiting with a partner from conception to birth, excluding those who began cohabiting with the child’s father within 6 months postbirth. We excluded births to same-sex couples (*n* = 849), as these are not captured in the cohabitation register and may involve different mechanisms [32]. For further minor exclusions (*n* = 4548), see Supplementary Box S1.

### Birth outcomes

We analysed five birth outcomes: LBW (<2500 g), preterm birth (<37 weeks of gestation), small for gestational age (SGA, sex-stratified birthweight less than the 10th percentile of all singleton infants born in Finland in 1996–2020 within the gestational week), gestational-age-adjusted birthweight (in grams) and gestational age (in days). Given the changes in prevalence, access, and regulations surrounding MAR in Finland over the 25-year observation period, we adjusted all models for the 5-year intervals (1996–2000, 2001–05, 2006–10, 2011–15, 2016–20).

### Covariates

First, we controlled for a set of variables related to maternal health and socio-economic status. We controlled for maternal age (20–24, 25–29, 30–34, 35–39, and 40+), level of education (basic, secondary, and tertiary), and income (quintiles of the households’ equivalent disposable income prebirth). We additionally included whether the mother smoked during pregnancy as an important risk factor for children’s health [33, 34]. We adjusted for maternal mental health, as studies suggest that women undergoing MAR often have a higher prevalence of mental ill-health preconception [23] which may affect birth outcomes [35–37]. To construct a measure of a woman’s mental health up to 5 years pre-MAR, we used data on purchases of anxiolytics (ATC codes N05B), hypnotics and sedatives (N05C), and antidepressants (N06A), drugs often used to treat anxiety, depression, insomnia, and related mental health conditions. We grouped women as purchasers (≥1) and nonpurchasers within this timeframe, considering only purchases from age 18 onwards. Second, we accounted for multiplicity (twins/triplets), which are more prevalent in MAR pregnancies due to treatment procedures and could mediate the association between MAR and birth outcomes [2–5].

### Statistical analysis

We used logistic models for binary outcomes, estimating three regressions for each outcome (LBW, preterm, and SGA). We used linear models with robust standard error for gestational age and variance-weighted least squares linear regression for birthweight, incorporating fractional polynomial terms [38, 39] to adjust for gestational age (see Supplementary Box S2 for further details). Model 1 presents the baseline associations controlling only for period and child’s sex. Model 2 introduces controls for maternal characteristics (age at birth, level of education, income, smoking during pregnancy, and history of psychotropic medication). Model 3 (fully adjusted) adds controls for multiplicity.

For sensitivity analyses, we restricted the sample to singletons to remove the influence of multiple births and examined the period 2006–20, when births to single MAR mothers were most common.

We also present additional analysis for SGA excluding infants born before 28 weeks’ gestation and restricting the outcome to term-SGA (≥37 weeks).

## Results

MAR treatments were utilized in 7.8% (*n* = 41 670) of pregnancies; among MAR mothers, 5.4% conceived while single, versus 7.5% of SC mothers. The share of single mothers among first-time MAR rose to 10.2% in 2016–20, while remaining stable at 7%–8% for spontaneous conceptions (Fig. 2).

**Figure 2:**
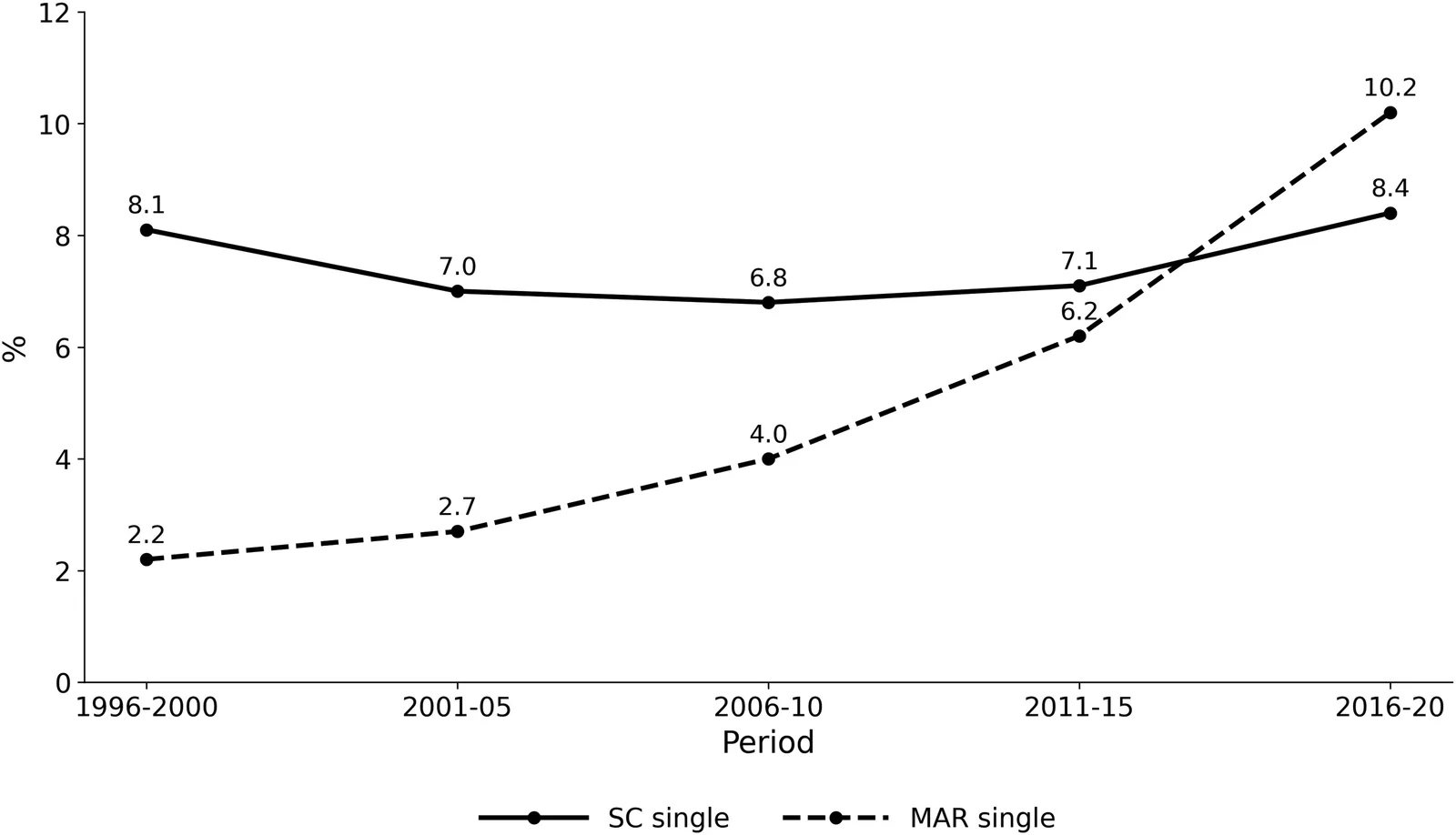
Proportion of single women among first-time mothers in Finland across five periods (1996–2000 to 2016–20), comparing spontaneous conception (SC: solid line) and medically assisted reproduction (MAR: dashed line).

Children of single MAR mothers had, on average, a higher proportion of LBW and preterm births compared to SC children in both groups (Table 1). However, SGA was less common than among children of single SC mothers. They also had, on average, lower birthweight and shorter gestational age than SC children. Among MAR children, those born to single mothers had, on average, better outcomes than those born to partnered mothers. In contrast, among SC children, those born to partnered mothers had the lowest prevalence of adverse birth outcomes.

**Table 1:** Descriptive statistics of birth outcomes of first-born children in Finland, 1996–2020, by mode of conception and maternal partnership status.^a^

	MAR single	SC single	MAR partnered	SC partnered
Birth outcomes	(*n* = 2230)	(*n* = 36 352)	(*n* = 39 350)	(*n* = 451 295)
Low birthweight (%), <2500 g	8.0	5.7	9.7	4.3
95% CI	(6.7–8.9)	(5.5–5.9)	(9.4–10.0)	(4.3–4.4)
Preterm (%), <37 weeks	10.0	6.2	11.8	5.5
95% CI	(8.3–10.7)	(6.0–6.5)	(11.5–12.1)	(5.4–5.6)
Small for gestational age (%)	9.9	12.9	10.8	9.9
95% CI	(8.6–11.1)	(12.5–13.2)	(10.5–11.1)	(9.8–9.9)
Birthweight (grams)	3353	3383	3313	3436
95% CI	(3326–3379)	(3377–3389)	(3306–3319)	(3434–3438)
Gestational age (weeks)	39.0	39.4	38.8	39.4
95% CI	(38.9–39.1)	(39.3–39.4)	(38.8–38.8)	(39.4–39.4)

a 95% confidence intervals are presented underneath the mean statistics. Mean birthweight is presented across the full gestational age distribution for descriptive comparability across groups, whereas the regression analyses model birthweight adjusted for gestational age. ‘MAR’ refers to medically assisted reproduction, ‘SC’ stands for spontaneous conception.

Source: Authors’ calculations from Finnish population register data.

The demographic profile of single MAR mothers differed notably from other groups of mothers (Table 2). On average, they were 3 years older than partnered MAR mothers and 7.5 years older than SC mothers. Education levels were highest among single MAR mothers (66.8% with tertiary education), followed closely by partnered MAR mothers (64.3%), while single SC women had the lowest education levels (22.7%). Income distribution for single MAR mothers was comparable to partnered SC mothers. Partnered MAR women had the highest average income, while single SC mothers had the lowest. Smoking during pregnancy was less prevalent among MAR mothers (6.9% single, 5.3% partnered), with single SC women reporting the highest proportion of smoking (35.2%). Single women were more likely to have used psychotropic medication within 5 years preconception (28.3% of single MAR mothers and 22.7% of single SC mothers) compared to partnered women in both groups. MAR-conceived children were more likely to be multiple births.

**Table 2:** Characteristics of women giving first birth in Finland, 1996–2020, and their children, by mode of conception and maternal partnership status.

	MAR single	SC single	MAR partnered	SC partnered	All
Woman’s age at birth (%)					
20–24	4.5	39.1	4.5	24.7	24.1
25–29	12.2	27.0	26.0	39.6	37.6
30–34	24.0	19.3	40.1	26.3	26.9
35–39	34.3	11.2	22.9	8.1	9.5
40+	24.9	3.4	6.5	1.3	1.9
Woman’s mean age at birth, (SD)	35.7 (5.7)	27.9 (5.9)	32.5 (4.7)	28.5 (4.7)	28.8 (4.9)
Period					
1996–2000	7.6	21.7	19.5	19.8	19.8
2001–05	7.9	19.5	16.2	20.7	20.2
2006–10	15.4	20	20.7	22.2	21.9
2011–15	25.6	20	22.1	20.9	20.9
2016–20	43.5	18.8	21.6	16.4	17.1
Educational level					
Basic	8.1	31.6	5.6	12.6	13.4
Secondary	25.1	45.7	30.1	43.2	42.4
Tertiary	66.8	22.7	64.3	44.2	44.3
Income quintile (year prebirth)					
1st quintile	9.1	33.1	4.0	13.8	14.4
2nd quintile	10.0	18.4	5.2	12.2	12.1
3rd quintile	16.6	16.8	9.4	15.2	14.8
4th quintile	26.2	13.3	21.7	22.4	21.8
5th quintile	33.0	9.9	56.3	30.0	30.6
Missing	5.0	8.6	3.3	6.3	6.3
Smoking during pregnancy					
No	90.4	61.8	92.3	83.4	82.6
Yes	6.9	35.2	5.3	14.3	15.0
Unknown	2.7	3.1	2.3	2.3	2.4
Psychotropic medicine purchases within 5 years pre-MAR (%)					
Yes	28.3	22.7	15.2	13.0	13.9
Multiplicity (%)					
Twins and triplets	6.1	1.1	8.6	1.0	1.6
Child’s sex: female (%)	50.7	48.9	48.9	48.8	48.8
Total *N*	2230	36 352	39 350	451 295	529 227

SD, standard deviation. ‘MAR’ refers to medically assisted reproduction, ‘SC’ stands for spontaneous conception.

Source: Authors’ calculations from Finnish population register data.

Table 3 presents coefficients for all birth outcomes among children of single MAR, single SC, partnered MAR, and partnered SC mothers. To help visualize the results, Fig. 3 shows the odds ratios (ORs) for LBW, preterm birth, and SGA in Models 1 and 3.

**Figure 3:**
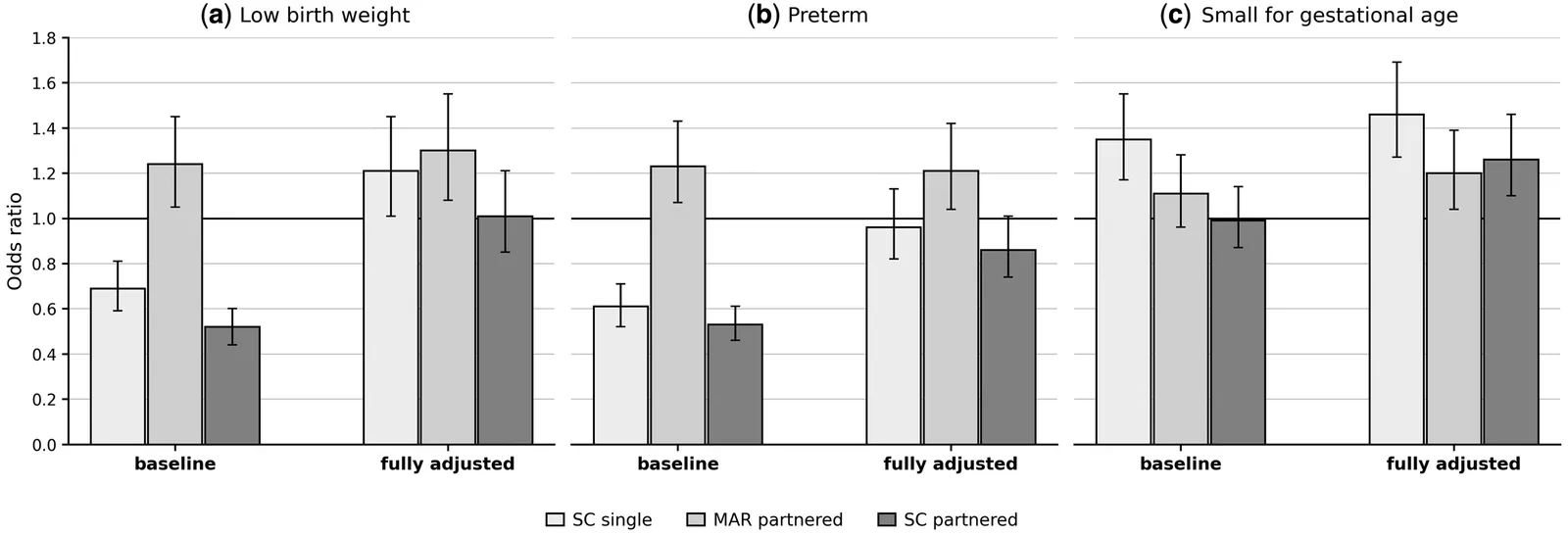
Odds ratios and 95% confidence intervals for (a) low birthweight, (b) preterm birth, and (c) small for gestational age for first-born children in Finland, 1996–2020, by mode of conception and maternal partnership status. Estimates are shown for unadjusted (baseline) and fully adjusted models. ‘MAR’ refers to medically assisted reproduction, ‘SC’ stands for spontaneous conception. The reference category (solid line) is children born to single mothers who conceived through MAR. Baseline model controls only for period and child’s sex and presents the unadjusted descriptive association between MAR and low birthweight (a), preterm birth (b), and small for gestational age (c). Fully adjusted models are controlled for maternal age, level of education, income, smoking during pregnancy, psychotropic medication use 5 years pre-MAR, and multiple birth.

**Table 3:** Models of birth outcomes (95% CI) of first-born children in Finland, 1996–2020, by mode of conception and maternal partnership status: low birthweight, preterm birth, small for gestational age, gestational age, and birthweight.^a^

	Model 1 (baseline)	Model 2 (Model 1 + maternal age + level of education + income + smoking during pregnancy + psychotropic medication use 5 years pre-MAR)	Model 3 (Model 2 + multiple birth)
Low birthweight: odds ratios from logistic models			
SC single (ref: MAR single)	0.69 (0.59 to 0.81)	0.77 (0.66 to 0.91)	1.21 (1.01 to 1.45)
MAR partnered	1.24 (1.05 to 1.45)	1.42 (1.21 to 1.66)	1.30 (1.08 to 1.55)
SC partnered	0.52 (0.44 to 0.60)	0.65 (0.55 to 0.76)	1.01 (0.85 to 1.21)
Preterm: odds ratios from logistic models			
SC single (ref: MAR single)	0.61 (0.52 to 0.71)	0.69 (0.59 to 0.80)	0.96 (0.82 to 1.13)
MAR partnered	1.23 (1.07 to 1.43)	1.32 (1.14 to 1.53)	1.21 (1.04 to 1.42)
SC partnered	0.53 (0.46 to 0.61)	0.61 (0.53 to 0.71)	0.86 (0.74 to 1.01)
Small for gestational age: odds ratios from logistic models			
SC single (ref: MAR single)	1.35 (1.17 to 1.55)	1.34 (1.16 to 1.55)	1.46 (1.27 to 1.69)
MAR partnered	1.11 (0.96 to 1.28)	1.24 (1.08 to 1.43)	1.20 (1.04 to 1.39)
SC partnered	0.99 (0.87 to 1.14)	1.16 (1.00 to 1.33)	1.26 (1.10 1.46)
Gestational age (in days): coefficients from linear models to with robust standard errors			
SC single (ref: MAR single)	3.0 (2.3 to 3.7)	2.5 (1.7 to 3.2)	1.1 (0.5 to 1.8)
MAR partnered	−1.1 (−1.8 to −0.4)	−1.4 (−2.2 to −0.7)	−0.9 (−1.5 to −0.3)
SC partnered	3.4 (2.7 to 4.2)	2.7 (2.0 to 3.5)	1.4 (0.7 to 2.0)
Birthweight (in grams): coefficients from variance − weighted least squares linear regression with fractional polynomial adjustment for gestational age			
SC single (ref: MAR single)	−49 (−67 to −31)	−52 (−70 to −34)	−67 (−85 to −49)
MAR partnered	−14 (−32 to 3)	−30 (−48 to −12)	−24 (−42 to −6)
SC partnered	−7 (−24 to 11)	−30 (−47 to −12)	−45 (−62 to −27)

a 95% CI are presented in parentheses. Baseline models control for period and child’s sex. ‘MAR’ refers to medically assisted reproduction, ‘SC’ stands for spontaneous conception.

Source: Authors’ calculations from Finnish population register data.

In Model 1, children of single MAR mothers had a higher risk of LBW and preterm birth and lower gestational age than SC children in both groups, but a lower risk than children of partnered MAR mothers. Differences in SGA and gestational-age-adjusted birthweight between children of single MAR, partnered MAR, and partnered SC groups were relatively small in the unadjusted models, although children of single MAR mothers had lower risks of SGA and higher gestational-age-adjusted birthweight than those of single SC mothers. After adjusting for maternal socio-demographic and health characteristics (Model 2), differences in LBW, preterm birth, and gestational age between children of single MAR mothers and SC children in both groups were largely attenuated, while the relative advantage of single MAR mothers became more pronounced for SGA and gestational-age-adjusted birthweight. For example, the OR for LBW among children of single SC mothers increased from 0.69 [95% confidence interval (CI) 0.59–0.81] to 0.77 (95% CI 0.65–0.91), and among children of partnered SC mothers, the OR increased from 0.51 (95% CI 0.44–0.60) to 0.64 (95% CI 0.55–0.75), relative to children of single MAR mothers (reference). In contrast, the odds of LBW among children of partnered MAR mothers increased from 1.24 (95% CI 1.05–1.45) in Model 1 to 1.41 (95% CI 1.21–1.66) in Model 2 compared with children of single MAR mothers.

After additional adjustment for multiplicity (Model 3), differences in LBW, preterm birth, and gestational age between children of single MAR and partnered SC mothers were small, with overlapping confidence intervals for binary outcomes (e.g. OR LBW = 1.01; 95% CI 0.85–1.21). In contrast, differences in LBW relative to children of single SC mothers increased in magnitude and reversed in direction, indicating poorer outcomes for the latter (OR LBW = 1.21; 95% CI 1.01–1.45), while risks of preterm birth were similar (OR = 0.96; 95% CI 0.82–1.13). For SGA and gestational-age-adjusted birthweight, the relative advantage of single MAR mothers became more pronounced compared with both SC groups (e.g. OR SGA = 1.26; 95% CI 1.10–1.46 relative to partnered SC mothers and OR = 1.46; 95% CI 1.27–1.69 relative to single SC mothers). Differences for children of partnered MAR mothers attenuated but remained pronounced across all outcomes.

Findings from the fully adjusted models for all outcomes were also highly similar when multiple births were excluded from the analytical sample (Supplementary Table S1). Sensitivity analyses for SGA excluding infants born before 28 weeks’ gestation and restricting the outcome to term-SGA (≥37 weeks) produced similar results (Supplementary Table S2). Additional sensitivity analyses (Model 3 specification) excluding births before 2006 (Supplementary Table S3), when access to treatments was more limited for single women in the public sector, are in line with the main analyses. However, the relative advantage (e.g. differences in OR for LBW) of children of single MAR mothers in comparison to those of single SC and partnered MAR mothers was more pronounced in these analyses.

## Discussion

### Main findings and contributions

Using Finnish register data, we investigated birth outcomes among children of single MAR mothers. In unadjusted analyses, they had higher risks of LBW and preterm birth and lower gestational age than children of single and partnered SC mothers, but better outcomes than children of partnered MAR mothers. Patterns for SGA and gestational-age-adjusted birthweight were more favourable, with children of single MAR mothers showing outcomes similar to those of partnered SC mothers and better than those of single SC mothers. After accounting for maternal characteristics and multiplicity, differences in LBW, preterm birth, and gestational age between children of single MAR and partnered SC mothers were largely attenuated, while a relative advantage in SGA and gestational-age-adjusted birthweight remained. In contrast, children of single SC and partnered MAR mothers exhibited poorer outcomes. Three main implications emerge from the results.

First, MAR treatments per se are unlikely to play a major role in determining birth outcomes, as adjusted results show children of single MAR mothers have similar outcomes to those of partnered SC mothers and better outcomes than those of partnered MAR mothers. The latter difference likely reflects underlying subfertility among partnered MAR mothers, a known risk factor for adverse birth outcomes. This aligns with research showing that groups who use MAR without subfertility—such as lesbian couples—have birth outcomes similar to those of spontaneously conceiving [32].

Second, the high prevalence of multiple births among MAR-conceived children accounts for the largest share of the elevated risks observed in unadjusted analyses among children of single MAR mothers. This is supported by the sequential modelling results: differences in LBW, preterm birth, and gestational age attenuate substantially after adjustment for multiplicity (Model 3), while the favourable results for SGA and adjusted-for-gestational-age birthweight become more pronounced, indicating that multiple births account for a substantial share of the excess risks. Supplementary analyses restricted to singleton births further support this interpretation, as unadjusted differences between children of single MAR and SC mothers are markedly reduced when multiple births are excluded (Supplementary Table S1). Moreover, a more pronounced relative advantage compared to children of single SC and partnered MAR mothers in recent years (2006–20; Supplementary Table S3) coincides with a marked decline in multiple birth rates in this group, from 12.4% in 1996–2000 to 3.6% in 2016–20 (Supplementary Table S4). This trend likely reflects changes in clinical practice, particularly the increasing adoption of elective single embryo transfer [40], as well as regulatory developments. Taken together, these findings highlight the importance of reducing multiple births to improve perinatal outcomes in this population.

Third, the results challenge generalized assumptions about single motherhood. While single motherhood has often been linked to lower socioeconomic status and negative outcomes for children [9–12], this pattern may not apply to single MAR mothers. Single mothers ‘by choice’ represent a positively selected group, characterized by strong fertility intentions, sufficient financial resources, an extensive support network and emotional resilience to raise a child independently, partly distinguishing them from single mothers who conceive spontaneously [41–43]. In contrast, single mothers who conceive spontaneously are a more heterogeneous group and include women who may not have intended the pregnancy and who are more likely to face socioeconomic disadvantage [44]. The poorer outcomes observed among single SC births likely reflect this compositional disadvantage. Taken together, these findings highlight the importance of distinguishing between different pathways into single motherhood and caution against treating single mothers as a homogeneous group.

This study contributes novel population-level evidence on an emerging and understudied group. As delayed childbearing, partnership instability [29] and access to MAR increase, single motherhood via MAR is likely to become more common. While many women in this group report that they would have preferred to have children within a partnership, constraints in partner availability and biological timing often shape their pathways to parenthood [8, 25, 28, 45]. Our study examined a context where MAR treatments are largely publicly subsidized, resulting in a more diverse population of women accessing these services compared to countries with predominantly private funding. However, we believe that the observed associations may be generalizable to other contexts as social acceptance grows and single women’s treatment access expands globally [46].

### Strengths and limitations

Our study’s strengths include the use of high-quality population register data that allow us to compare birth outcomes of children of single MAR mothers to those of single SC mothers and partnered MAR and SC mothers. These comprehensive population-level data avoid nonresponse and recall biases of survey data. We employed all available data sources to comprehensively identify children conceived through MAR, determine maternal partnership status at conception/birth, and capture maternal sociodemographic characteristics and mental health.

Our study has a few limitations. As the cohabitation register does not allow identifying same-sex or living-apart-together couples, it is possible that some women classified as single had partners. Nevertheless, we believe that these couples only represent a few cases and will not affect the overall results from the analyses, as we excluded identifiable same-sex couples, and the prevalence of living-apart-together couples is low among new parents, as such partnerships tend to either dissolve or transition to cohabitation shortly after conception [47–49]. To further minimize misclassification, we considered women who started living with the child’s father within 6 months postbirth as partnered [48]. Our data could not differentiate treatment types (e.g. OI/AI/IVF) which are known to affect birth outcomes [2, 5]. However, due to potential underreporting of less invasive treatments in our data and single women’s greater likelihood of undergoing IVF [8], our sample likely over-represents more invasive treatments, making our estimates comparing birth outcomes among single and partnered MAR women conservative. Although we adjusted for a range of sociodemographic and maternal health characteristics, some potentially relevant confounders were not available in the register data. For example, we lacked information on health behaviours and psychosocial factors such as substance use, nutrition, or stress and social support, which may differ by partnership status and route to parenthood. The proxy for mental health based on medication purchases may also miss untreated or nonpharmacologically treated conditions. While residual confounding due to these unobserved factors cannot be ruled out, we do not expect that such biases would change our conclusions regarding single MAR mothers. If anything, unobserved characteristics among single MAR mothers—such as stronger planning, health behaviours, or access to resources—are likely to be protective rather than detrimental, suggesting that any remaining bias would, if anything, lead us to underestimate rather than overstate their relative advantage.

## Conclusions

Children of single mothers ‘by choice’ had, on average, more favourable birth outcomes than those of single SC and partnered MAR mothers, and similar outcomes to partnered SC mothers. Although unadjusted analyses suggested increased risks of adverse birth outcomes, these differences appear to be largely attributable to higher rates of multiple births in the single MAR group and unlikely to be related to family structure or MAR treatments per se.

## Ethics approval

This study was approved by the Ethics Committee of Statistics Finland’s permission TK-52–1121-18.

## Supplementary Material

dyag172_Supplementary_Data

## Data Availability

The data that support the findings of this study are available from Statistic Finland. Restrictions apply to the availability of these data, which were used under license for this study.
