# Supplementary material for: Birth outcomes in single motherhood ‘by choice’: a register study of children conceived through medically assisted reproduction
Source: Int J Epidemiol. 2026 Sep 23;55(5):dyag172. doi: 10.1093/ije/dyag172 (PMC13653600; doi:10.1093/ije/dyag172)
Supplement: Supplementary file 1 [file dyag172_supplementary_data.zip › Supplementary_material_for_production_updt.docx]

[**Supplementary material**](https://academic.oup.com/ije/pages/General_Instructions#Supplementary%20material)

**Supplementary Box S1. Further detail on identification of single mothers in the Finnish register data**

We also excluded a small number of women who were single at conception but were living with a male partner other than the child’s father (n=876) at the time of birth as it is difficult to categorise these women as single or partnered. In cases where the mother separated from the father within 3 months post-conception and remained single until birth (n=2695, predominantly SC (97%)), we identified them as single as it is likely that the actual separation occurred earlier. For separations occurring between 3 months of conception and birth (n=3672, 97% SC), we excluded those women from the analysis as separation at later stages of pregnancy could negatively affect birth outcomes (Frimmel & Pruckner 2014) complicating the comparison to women who were single at conception and decided to have a child on their own (both MAR and SC).

Reference: Frimmel W, Pruckner GJ. Birth weight and family status revisited: evidence from Austrian register data. Health Econ 2014;23:426–45. <https://doi.org/10.1002/hec.2923>

**Supplementary Box S2. Further detail on linear models used for continuous outcomes**

For birthweight models adjusted for gestational age, we assessed the functional form of the association between gestational age and birthweight using scatterplots with smoothed fitted curves and residual plots from linear models. These diagnostics suggested departure from linearity, consistent with evidence that fetal growth does not progress linearly across gestation, particularly at earlier and later gestational ages.

Accordingly, birthweight models adjusted for gestational age were estimated using fractional polynomial specifications (Royston & Altman, 1994; Royston & Sauerbrei, 2008). Unlike standard linear adjustment, fractional polynomials allow the slope to change across gestational age, flexibly modelling the non-linear relationship between gestational age and birthweight. We used a two-term fractional polynomial specification, which allows gestational age to enter the model through two selected power transformations, providing a parsimonious but flexible adjustment for gestational duration. Following estimation of the fractional polynomial models, standard regression diagnostics were assessed using residual-versus-fitted plots, Q–Q plots, and formal tests for heteroskedasticity, including the Breusch-Pagan/Cook-Weisberg and White tests. Residual diagnostics suggested that the fractional polynomial specification adequately captured the non-linear association between gestational age and birthweight, although evidence of heteroskedasticity remained. We therefore used variance-weighted least squares estimation, weighting observations by the inverse residual variance, to improve efficiency and reduce the influence of observations with greater variability.

For gestational age models, we evaluated standard linear regression assumptions by inspecting the distribution of gestational age, regression residuals, residual-versus-fitted plots, Q–Q plots, and formal tests for heteroskedasticity (Breusch-Pagan/Cook-Weisberg and White tests). These diagnostics suggested some departures from normality and constant variance, with evidence of heteroskedasticity (p<0.001). We therefore estimated gestational age models using heteroskedasticity-robust standard errors to obtain valid statistical inference without explicitly modelling the variance structure.

**Supplementary Table S1. Birth outcomes (95% CI) of first-born children in Finland, 1996-2020, by mode of conception and maternal partnership status: a) low birthweight; b) preterm birth; c) small for gestational age; d) gestational age; e) adjusted-for-gestational-age birthweight. Singletons only**

**Birth outcomes**

**Baseline model	Fully adjusted model**

| a) Low birthweight. Odds ratios from logistic models | | |
| --- | --- | --- |
| SC single (Ref: MAR single) | 1.06 (0.86, 1.31) | 1.22 (0.99, 1.50) |
| MAR partnered | 1.21 (0.98, 1.48) | 1.43 (1.16, 1.76) |
| SC partnered | 0.78 (0.63, 0.95) | 1.01 (0.83, 1.24) |
| b) Preterm. Odds ratios from logistic models | | |
| SC single (Ref: MAR single) | 0.81 (0.68, 0.96) | 0.92 (0.77, 1.10) |
| MAR partnered | 1.14 (0.96,1.36) | 1.25 (1.05, 1.49) |
| SC partnered | 0.70 (0.59, 0.83) | 0.82 (0.69, 0.98) |
| c) Small for gestational age. Odds ratios from logistic models | | |
| SC single (Ref: MAR single) | 1.52 (1.30, 1.77) | 1.51 (1.29, 1.77) |
| MAR partnered | 1.11 (0.95,1.30) | 1.25 (1.07, 1.46) |
| SC partnered | 1.12 (0.96, 1.30) | 1.30 (1.11, 1.51) |
| d) Gestational age (in days). Coefficients from linear models, with robust standard errors | | |
| SC single (Ref: MAR single) | 1.6 (0.9, 2.3) | 1.1 (0.4, 1.8) |
| MAR partnered | -0.6 (-1.2, 2.3) | -1.0 (-1.6, -0.3) |
| SC partnered | 2.0 (1.3, 2.6) | 1.4 (0.7, 2.0) |
| e) Birthweight (in grams). Coefficients from variance-weighted least squares linear regression with fractional polynomial adjustment for gestational age | | |
| SC single (Ref: MAR single) | -65 (-83, -46) | -67 (-86, -49) |
| MAR partnered | -9 (-28, 9) | -25 (-43, -6) |
| SC partnered | -22 (-40, -4) | -45 (-63, -27) |

*Note*: ‘MAR’ refers to medically assisted reproduction, ‘SC’ stands for spontaneous conception. 95% CI are presented in parentheses. Baseline models control for period and child’s sex. Fully adjusted models are controlled for period, child’s sex, maternal age, level of education, income, smoking during pregnancy, psychotropic medication use 5 years pre-MAR.

*Source*: Authors’ calculations from Finnish population register data.

**Supplementary Table S2. Birth outcomes (95% CI) of first-born children in Finland, 1996-2020, by mode of conception and maternal partnership status: a) small for gestational age excluding infants born before 28 weeks’ gestation; b) small for gestational age for term infants (>=37 weeks of gestation)**

|  | **Model 1 (baseline)** | **Model 2 (Model 1 + maternal age + level of education + income + smoking during pregnancy + psychotropic medication use five years pre-MAR)** | **Model 3 (Model 2 + multiple birth)** |
| --- | --- | --- | --- |
|  | a) Small for gestational age (excluding <28 weeks of gestation). Odds ratios from logistic models | | |
| SC single (Ref: MAR single) | 1.34 (1.16, 1.54) | 1.33 (1.15, 1.54) | 1.45 (1.26, 1.68) |
| MAR partnered | 1.10 (0.95, 1.27) | 1.23 (1.07, 1.42) | 1.19 (1.03, 1.38) |
| SC partnered | 0.99 (0.86, 1.13) | 1.14 (0.99, 1.32) | 1.25 (1.08, 1.44) |
|  | b) Small for gestational age (only 37+ weeks of gestation). Odds ratios from logistic models | | |
| SC single (Ref: MAR single) | 1.31 (1.13, 1.52) | 1.30 (1.12, 1.51) | 1.41 (1.21, 1.64) |
| MAR partnered | 1.07 (0.92, 1.24) | 1.19 (1.03, 1.39) | 1.16 (1.00, 1.35) |
| SC partnered | 0.96 (0.83, 1.11) | 1.11 (0.96, 1.28) | 1.20 (1.04, 1.40) |

*Note*: ‘MAR’ refers to medically assisted reproduction, ‘SC’ stands for spontaneous conception. 95% CI are presented in parentheses. Baseline models control for period and child’s sex.
*Source*: Authors’ calculations from Finnish population register data.

**Supplementary Table S3. Birth outcomes (95% CI) of first-born children in Finland, 2006-2020, by mode of conception and maternal partnership status: a) low birthweight; b) preterm birth; c) small for gestational age; d) gestational age; e) adjusted-for-gestational-age birthweight**

**Birth outcomes**

**Baseline model	Fully adjusted model**

|  | a) Low birthweight. Odds ratios from logistic models | |
| --- | --- | --- |
| SC single (Ref: MAR single) | 0.82 (0.68, 0.99) | 1.33 (1.08, 1.64) |
| MAR partnered | 1.25 (1.04, 1.50) | 1.42 (1.16, 1.75) |
| SC partnered | 0.59 (0.49, 0.71) | 1.07 (0.87, 1.31) |
|  | b) Preterm. Odds ratios from logistic models | |
| SC single (Ref: MAR single) | 0.70 (0.59, 0.83) | 1.03 (0.85, 1.25) |
| MAR partnered | 1.25 (1.06, 1.48) | 1.33 (1.11, 1.60) |
| SC partnered | 0.61 (0.52, 0.72) | 0.93 (0.78, 1.12) |
|  | c) Small for gestational age. Odds ratios from logistic models | |
| SC single (Ref: MAR single) | 1.41 (1.20, 1.65) | 1.53 (1.30, 1.79) |
| MAR partnered | 1.12 (0.96,1.32) | 1.24 (1.06, 1.46) |
| SC partnered | 1.05 (0.90, 1.22) | 1.30 1.11, 1.52) |
|  | d) Gestational age (in days). Coefficients from linear models, with robust standard errors | |
| SC single (Ref: MAR single) | 2.2 (1.4, 2.9) | 0.7 (0.0, 1.4) |
| MAR partnered | -1.1 (-1.9, -0.3) | -1.2 (-1.9, -0.5) |
| SC partnered | 2.7 (2.0, 3.4) | 1.1 (0.4, 1.8) |
|  | e) Birthweight (in grams). Coefficients from variance-weighted least squares linear regression with fractional polynomial adjustment for gestational age | |
| SC single (Ref: MAR single) | -52 (-72, -33) | -72 (-92, -52) |
| MAR partnered | -15 (-34, 5) | -28 (-47, -8) |
| SC partnered | -11 (-30, 8) | -47 (-66, -28) |

*Note*: ‘MAR’ refers to medically assisted reproduction, ‘SC’ stands for spontaneous conception. 95% CI are presented in parentheses. Baseline models control for period and child’s sex. Fully adjusted models are controlled for period, child’s sex, maternal age, level of education, income, smoking during pregnancy, psychotropic medication use 5 years pre-MAR.

*Source*: Authors’ calculations from Finnish population register data.	**Supplementary Table S4. Proportion of multiple births among of first-born children in Finland, 1996-2020, by mode of conception and maternal partnership status**

|  | 1996-2000 | 2001-2005 | 2006-2010 | 2011-2015 | 2016-2020 |
| --- | --- | --- | --- | --- | --- |
| MAR single | 12.4 | 10.7 | 10.5 | 4.6 | 3.6 |
| SC single | 1.1 | 1.1 | 1.0 | 0.9 | 1.2 |
| MAR partnered | 14.0 | 12.1 | 9.0 | 5.7 | 3.9 |
| SC partnered | 1.0 | 1.1 | 1.1 | 1.1 | 1.1 |

*Note*: ‘MAR’ refers to medically assisted reproduction, ‘SC’ stands for spontaneous conception.

*Source*: Authors’ calculations from Finnish population register data.
